# BRASH syndrome with a complete heart block- a case report

**DOI:** 10.1186/s12872-024-03782-6

**Published:** 2024-02-19

**Authors:** Habtamu Mesele Gebray, Abirham Eneyew Abeje, Abayneh Tunta Boye

**Affiliations:** 1 Weldia Comprehensive Specialized Hospital, Weldia, Ethiopia; 2 Weldia University, Weldia, Ethiopia

**Keywords:** BRASH, Hyperkalemia, Bradycardia, Syndrome, Shock, Cardiology

## Abstract

**Introduction:**

BRASH syndrome (Bradycardia, Renal failure, Atrioventricular (AV) nodal blocking agent, Shock and Hyperkalemia) is a recently emerging diagnosis that describes the profound bradycardia seen in patients on AV nodal blockers who present with acute kidney injury (AKI) and hyperkalemia.

**Case presentation:**

We present a case of a 68 years old female patient with past history of hypertension taking atenolol and Enalapril presented to emergency department with the complaint of loss of consciousness of 02 hours duration. She had 03 days history of fatigue, poor oral intake, decreased urine output, appetite loss, vertigo and global headache. Her vital signs were blood pressure of 60/40 mmHg, absent radial pulse and temperature of 36.4 °C. Her systemic examination was remarkable for dry buccal mucosa; apical heart rate was 22 beats per minute. Glasgow Coma Scale was 13/15. Her laboratory tests showed creatinine of 1.83 mg/dL, blood urea nitrogen of 89 mg/dL and potassium elevated to the level of 6.39 mEq/dL. ECG revealed complete heart block with a normal QT interval and T waves and no U waves with ventricular rate of 22 beats per minute. Her previous medications were discontinued and the patient was resuscitated with intravenous (IV) fluids. She was given 03 doses of 1 mg atropine every 5 minutes but there was no increment in heart rate. She was given 50% dextrose with 10 international units of regular insulin, 1 g of calcium gluconate and Intravenous perfusion of norepinephrine and dopamine. Subsequently, after 14 hours of ICU admission the patient had a cardiac arrest with asystole and resuscitation was attempted but she couldn’t survive.

**Conclusion:**

BRASH syndrome is largely an under-recognized life threatening clinical diagnosis. Physicians should have high index of suspicion for BRASH when they encounter patients with bradycardia, hyperkalemia, and renal failure, as timely diagnosis is crucial in the management.

## Introduction

The term BRASH syndrome (Bradycardia, Renal failure, Atrioventricular (AV) nodal blocking agent, Shock and Hyperkalemia) was first coined in 2016 by Dr. Josh Farkas. He proposed a pathophysiologic cycle of events leading to refractory bradycardia and renal dysfunction, which does not respond to the usual advanced cardiac life support (ACLS) [1]. BRASH syndrome has recently emerged as a diagnosis that describes the profound bradycardia seen in patients on AV nodal blockers who present with acute kidney injury (AKI) and hyperkalemia [2]. The pathophysiology of BRASH syndrome is derived from synergism between AV nodal blockade and hyperkalemia. A seemingly non harmful event such as mild dehydration causes a mild reduction in renal perfusion and reduced glomerular filtration rate. Cardiovascular medications, which are cleared through kidney, such as beta-blockers, non-dihydropyridine calcium channel blockers, and anti-arrhythmics begin to accumulate due to this insult. Continued accumulation induces renal hypoperfusion due to the resulting bradycardia and decreased cardiac output, which further precipitates the compounding renal failure. Renal failure can induce hyperkalemia and further decrease the excretion of drugs that are partially or fully renally cleared. Therapeutic doses of such agents generally do not cause severe bradycardia; however, with decreased renal clearance and hyperkalemia, the effects of AV nodal blockers are potentiated [3, 4]. It is a vicious cycle precipitated by renal failure, leading to hyperkalemia and accumulation of AV nodal blockers like beta-blockers (BB) or calcium channel blockers (CCB). The vicious cycle is often initiated by hypovolemia or AV-nodal-blocking medications and if not quickly diagnosed, it can progress to shock and multiple-organ failure, needing transvenous pacing and hemodialysis [5]. AV nodal blockers have been used widely to treat a wide range of health problems, such as hypertension and arrhythmia, but the recognition and characterization of BRASH as its own entity is rather newly evolving. The rapid institution of hemodynamic support, correction of hyperkalemia, and beta-blocker withdrawal is essential for the recovery of patients with BRASH syndrome without the need for renal replacement therapy [6]. This syndrome is an emerging clinical entity that can lead to catastrophic events if left untreated.

Here we present a case of BRASH syndrome.

## Case presentation

A 68 years old female patient with past medical history of hypertension presented to emergency department with the complaint of loss of consciousness of 02 hours duration. She was diagnosed with hypertension 02 years back and she was on conservative management for 01 month after which she was started on Enalapril 10 mg per day. After 6 months, Atenolol 50 mg daily was added due to associated sinus tachycardia. She had good control of her hypertension. Three days prior to her presentation, she started to have complaints of progressively worsening fatigue, poor oral intake, decreased urine output, appetite loss, vertigo and global headache. One day prior to her arrival, she had a single episode of watery diarrhea. Currently she presented with the complaint of loss of consciousness of 02 hours duration. The family stated that she was taking atenolol 50 mg daily and Enalapril 10 mg daily properly till the time of her presentation. Lately, she had poor adherence to her follow up visits and she missed her last 03 episodes of monthly appointments. She had no fever, chills, chest pain, dyspnea, or body swelling. No history of diabetes mellitus, renal or cardiac disease.

On evaluation, the patient was lethargic with blood pressure of 60/40 mmHg, absent radial pulse, cold extremities and temperature of 36.4 °C. Her examination was remarkable for dry buccal mucosa; apical heart rate was 22 beats per minute. On Central Nervous System examination, GCS (Glasgow Coma Scale) was 13/15 (4–4-5-best Eye response = 4/4, best verbal response = 4/5, best motor response = 5/6). The other system examinations were normal.

**Fig. 1:**
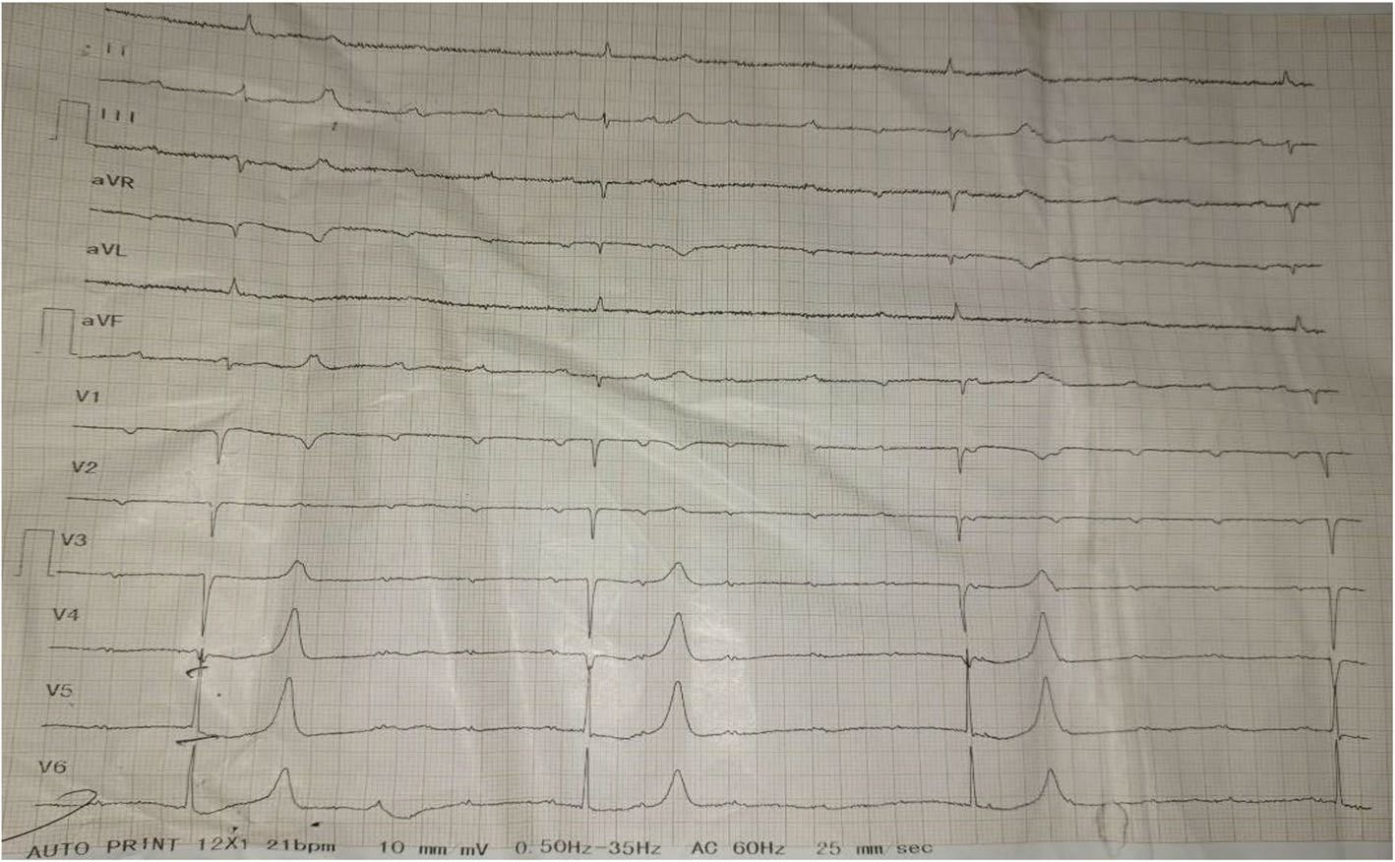
ECG of the patient showing complete heart block with severe bradycardia with heart rate of 22 beats per minute

The patient’s laboratory results were notable for elevation of creatinine at 1.83 mg/dL, blood urea nitrogen of 89 mg/dL and potassium elevated to the level of 6.39 mEq/dL. She had normal blood sugar level and complete blood count panel. She had normal TSH (thyroid stimulating hormone) level (Table 1). Urine analysis was nonrevealing. Troponin level was 0.1. ECG revealed complete heart block with a normal QT interval and T waves and no U waves with ventricular rate of 22 beats per minute (Fig. 1). Bedside point of care ultrasound showed normal findings. Bedside echocardiography showed ejection fraction of 53% with no apparent regional wall motion abnormality. Chest X-ray didn’t show any evidence of consolidation, pulmonary congestion or pleural effusion.

**Table 1:** Laboratory values from admission time and after 6 hours of ICU admission with reference ranges

Lab results	At admission	After 6 hours in ICU	Reference
Hgb (g/dL)	14.1	Not repeated	12.3–15.3
WBC (10*3/ul)	9.2	Not repeated	4.00–11.00
FBS (mg/dL)	84	80	70–100
Sodium (mmol/L)	138	135	135–145
Potassium (mmol/L)	6.39	6.01	3.5–5.5
iCalcium (mmol/L)	1.07	1.1	1.05–1.35
Chloride (mmol/L)	101.3	100	96–108
ALT (mg/dL)	53	48	0–40
AST (mg/dL)	40	36	0–40
ALP (mg/dL)	128	106	0–270
Urea nitrogen (mg/dL)	89	90	6–22
Creatinine (mg/dL)	1.83	1.9	0.3–1.3
Troponin I (ng/mL)	0.2	0.1	0.0–0.3
TSH (mIU/mL)	2.6	Not repeated	0.3–4.2
Free T4 (nmol/L)	88	Not repeated	66–181

*Hgb* Hemoglobin, *WBC* White blood cells, *ALT* Alanine aminotransferase, *AST* Aspartate aminotransferase, *ALP* Alkaline phosphatase

At the emergency department, her previous medications were discontinued and the patient was resuscitated with an intravenous (I.V.) bolus of 30 mL/kg of sodium chloride 0.9% was given but there was no response to the shock. Then the patient was transferred to intensive care unit (ICU) for further management.

In the ICU, with the assessment of Cardiogenic shock secondary to symptomatic bradycardia 2ry to 3rd degree AV block and Electrolyte disturbance (moderate hyperkalemia) secondary to AKI (Acute Kidney Injury) she was given 03 doses of 1 mg atropine every 5 minutes but there was no increment in heart rate. Few hours later, the assessment of BRASH (Bradycardia, Renal failure, AV nodal blockage, Shock and Hyperkalemia) syndrome was considered and cardiology and nephrology teams were consulted. After re-evaluation, the following regimens were administered. She was given 10mls of 10% calcium gluconate slowly over 20 minutes mixed in 100 ml of glucose 5% for immediate antagonism of the cardiac effects of hyperkalemia. It raises the action potential threshold and reduces excitability, without changing the resting membrane potential. By restoring the difference between resting and threshold potentials and hence reversing the depolarization blockade due to hyperkalemia, intravenous calcium serves to protect the heart. Then the team decided to use medications with the effect of rapid reduction in plasma potassium concentration by redistribution into cells. Hence then she was given 10 international units of regular insulin intravenous with 25 g of glucose (03 vials of 40% glucose) intravenously over 15 minutes. This drives potassium into cells. After administration of these two drugs, there was no improvement in the vital signs or on the ECG tracing on the monitor. Latter, decision to initiate inotropes was made with close monitoring. Initially started with Dopamine at a dose of 5 mcg/kg/min by diluting 400 mg dopamine in 250 ml and escalated every 10 minutes and when reached to 20 mcg/kg/min, Norepinephrine was added. We initiated norepinephrine at a dose 0.01 mcg/kg/min and escalated to its maximum dose 2 mcg/kg/min. After 6 hours of this treatment, there was no improvement on the vital signs. The ECG was repeated and showed no change from the initial record. In the meantime, since there is no cardiac pacemaker placement service in our hospital, arrangement of transportation to a center where she could get transvenous pacemaker was in progress. Subsequently, after 14 hours of ICU admission the patient had a cardiac arrest with asystole and resuscitation was attempted but she couldn’t survive.

## Discussion

Transient abnormal rhythms have been reported due to many causes; however with our patient’s other abnormal lab findings, the persistence of bradycardia raised concerns for different etiologies. BRASH syndrome is an under recognized pathophysiological phenomenon. Although the pathophysiology underlying BRASH syndrome has been established since the 1990s, its recognition as a specific entity is very recent and little is known regarding its true epidemiology [5]. Older patients with multiple comorbidities, mainly cardiac and renal diseases, may be at higher risk of developing BRASH, especially if their medications include multiple different AV-nodal blocking medications [1]. Different triggers are noted in recent reports, including medications such as Ranolazine and Bactrim, anaphylaxis, and even COVID-19. The hallmark mechanism of BRASH syndrome is a synergistic effect of AV nodal blocking medications and hyperkalemia promoting bradycardia. This, in conjunction with renal injury, produces the cycle of objective findings that define BRASH syndrome [1]. Patients may present with a variety of non-specific signs and symptoms including symptomatic bradycardia, syncope, generalized weakness, altered mental status, dyspnea or dizziness/lightheadedness, and others, which makes diagnosis of BRASH syndrome very challenging [2, 7]. It is important for clinicians to maintain a broad differential diagnosis when considering BRASH syndrome, especially since the symptomatology overlaps with other conditions, including medication overdose causing AV node toxicity and secondary causes of hyperkalemia [8]. While an AV nodal blocker overdose can mimic the bradycardia and shock seen in BRASH syndrome, the clinical history can aid in distinguishing the two disease states since patients with BRASH syndrome typically report taking their medications as prescribed [1].

There are many possible differential diagnoses in our patient presenting with generalized weakness and confusion, dry mucous membranes, and bradycardia and poor oral intake. Our differential diagnosis ranges from simple dehydration causing hyponatremia, pre-renal acute kidney injury (AKI) to medication-induced, infections, hypothyroidism, congestive heart failure, and even acute coronary syndrome. Since she was afebrile, had a normal chest X –ray, and had a normal white blood cell count and normal urine analysis, infectious etiology was quickly ruled out. Initial ECG without any ischemic changes and decreased troponin levels ruled out an acute ischemic coronary event. AKI was thought to be caused by dehydration and worsened by her medication-Enalapril. Although these findings pointed to AKI and hyperkalemia as potential causes of the patient’s weakness and bradycardia, significant potassium elevation (typically > 7) is required for pure hyperkalemia to cause bradycardia of this magnitude. The persistence of shock despite our intervention in our patient can be well explained by the pathophysiology of BRASH syndrome; her initial dehydration leading to decreased renal perfusion which then results in decreased clearance of beta blockers from the body which potentiate its effect to induce decreased cardiac output or shock. Furthermore, bradycardia and Atenolol use raised the possibility of beta-blocker toxicity; however, the patient’s family reported medication compliance and denied overuse of her beta-blocker medication. As a result, neither hyperkalemia nor beta-blocker toxicity was likely to be the only cause of this patient’s symptoms, making BRASH syndrome the most likely cause in our case.

One of the common errors in managing BRASH syndrome is focusing only on a single component of the syndrome, rather than approaching it from its complex perspective [1]. Mild cases of BRASH syndrome frequently respond to simple medical treatments like intravenous calcium and fluid resuscitation. In moderate cases, the treatment of BRASH syndrome focuses on aggressive hyperkalemia therapy and fluid resuscitation. Hyperkalemia can be managed by membrane stabilization, shifting potassium intracellularly, and kaliuresis or dialysis. Fluid resuscitation depends on the volume status. As suggested by Dr. Farkas, isotonic bicarbonate is a good initial choice for fluid resuscitation, as most patients with BRASH syndrome will have a combination of hyperkalemia and metabolic acidosis. The traditional bradycardia management using the Advanced Cardiovascular Life Support (ACLS) algorithm, including atropine and cardiac pacing, may not succeed in patients with BRASH syndrome in more severe cases of shock [1, 9]. Most patients respond well to these general management principles without the need for more aggressive therapies. If these measures are not successful, then more advanced therapies should be considered. These include hemodialysis, IV lipid emulsions, IV glucagon and transdermal or transvenous cardiac pacing [9, 10]. Involvement of multidisciplinary team including specialists in cardiology and nephrology, in the management of BRASH syndrome, is highly encouraged. It is crucial in saving the life of affected patients. In our case, discontinuation of atenolol, administration of calcium gluconate, giving insulin with dextrose and administration of vasopressors like norepinephrine and dopamine with the involvement of different disciplines was unable to break the cycle of BRASH syndrome. And finally, Cardiac resuscitation following the ACLS protocols was not successful. This could raise the impression that the vicious cycle of BRASH syndrome might be irreversible after it reaches at a certain point. The next best option would have been giving transvenous pacing and hemodialysis.

Since it is a recently diagnosed entity, understanding the pathophysiology and early identification of BRASH has significant role in reducing mortality. Aggressive management of dehydration, electrolyte imbalance and shock is a critical measure in the management of BRASH. Early recognition of the syndrome and aggressive treatment is one of the determinant factors. Especially in a low income country like ours, where having pacemakers and dialysis is like a luxury, early intervention could help in saving more lives.

## Conclusion

BRASH syndrome is largely an under-recognized life threatening clinical diagnosis. Physicians should have high index of suspicion for BRASH when they encounter patients with resistant and self-potentiating bradycardia, hyperkalemia, and renal failure, as timely diagnosis is crucial in the management. Variable clinical presentations pose a diagnostic challenge. Improving under-standing of diverse presenting signs and symptoms and the influence of other conditions can help physicians in faster recognition and better management of this syndrome, ultimately improving survival and patient outcomes. Further research is needed to create and establish effective triaging tools, consistent diagnostic criteria, and therapeutic guidelines to reduce the delay until appropriate treatment, unnecessary interventions, and complications related to its complicated pathophysiology and presentation.

### Challenges and limitations

BRASH syndrome is a life-threatening yet largely underdiagnosed condition. There was a big challenge to diagnose our patient with BRASH syndrome. Variable clinical presentations with limited literature and absence of standard guideline pose a great diagnostic challenge. We have acknowledged that lack of transvenous pacing and hemodialysis service in our hospital has a negative impact on the outcome of our treatment. We encourage physicians to keep a high index of suspicion for BRASH while treating patients with resistant bradycardia, hyperkalemia and renal failure, as a timely diagnosis and treatment are the cornerstones of better outcome in managing patients with BRASH.

One of the limitations of this report is the nature of the case report being retrospective design giving no chance to establish a cause-effect relationship. The other is since it is a case report from a single center; it may not be representative of the general population. These limitations might have a negative impact on the generalizability of the findings.

## Data Availability

No datasets were generated or analysed during the current study.
